# The effect of using the hip exoskeleton assistive (HEXA) robot compared to conventional physiotherapy on clinical functional outcomes in stroke patients with hemiplegia: a pilot randomized controlled trial

**DOI:** 10.1186/s42490-024-00082-0

**Published:** 2024-08-01

**Authors:** Hamed Mamipour, Seyed Ali Hoseini, Hossein Negahban, Ali Moradi, Amir Hojjati, Fariborz Rezaeitalab, Mohammadreza Torshizian, Arefeh Mehrali, Mohammad Parsa, Iman Kardan, Hamed Tabesh, Ebrahim Ghayem Hassankhani, Alireza Akbarzadeh

**Affiliations:** 1 Department of Physical Therapy, School of Paramedical and Rehabilitation Sciences, Mashhad University of Medical Sciences, Mashhad, Iran; 2 Orthopedic Research Center, Mashhad University of Medical Sciences, Mashhad, Iran; 3 Center of Advance Rehabilitation and Robotics Research (FUM-CARE), Mechanical Engineering Department, Ferdowsi University of Mashhad, Mashhad, Iran; 4 Department of Neurology, School of Medicine, Mashhad University of Medical Sciences, Mashhad, Iran; 5 Department of Physical Therapy, School of Rehabilitation, University of Social Welfare and Rehabilitation Sciences, Tehran, Iran; 6 Department of Medical Informatics, Mashhad University of Medical Sciences, Mashhad, Iran

**Keywords:** HEXA, Stroke, Rehabilitation, Gait, Exoskeleton – robotics

## Abstract

**Trial design:**

This study is a pilot randomized clinical trial aimed to investigate the effect of using Hip Exoskeleton Assistive (HEXA) robot compared to conventional physiotherapy on the quality of walking, disability, and quality of life of stroke patients with hemiplegia.

**Methods:**

In this study, 24 patients were randomly assigned to the intervention group (robotic physiotherapy with HEXA robot), or control group (conventional physiotherapy). In each session, both groups received 30 min of conventional physiotherapy including electrotherapy and conventional exercises, and then the intervention group did gait training for 30 min with the HEXA robot and the control group for 30 min without the HEXA robot. The treatment program was 12 sessions, 3 times a week. Before the 1st and after the 12th sessions, both groups were evaluated for walking quality, disability, and quality of life.

**Results:**

The results showed that the main effect of time was significant (*P* < 0.05) in all outcomes and patients in both groups achieved significant improvement in all outcomes after the intervention. The main effect of the group was also significant in the outcomes of 6MWT (*P* < 0.05) and TUG (*P* < 0.05), and the intervention group patients experienced more distance and speed in these two tests. This study was approved by the ethics committee of Mashhad University of Medical Sciences (IR.MUMS.FHMPM.REC.1400.079 dated 28th Jan 2022). The trial was registered with the clinical trials site of www.IRCT.ir (IRCT20210730052024N1) on January 28th 2022.

**Conclusion:**

It seems that the HEXA robot can effectively improve walking capacity and speed.

**Supplementary Information:**

The online version contains supplementary material available at 10.1186/s42490-024-00082-0.

## Introduction

After heart disease, cancer, unwanted accidents, and chronic respiratory disease, stroke is the fifth leading cause of death and the leading cause of serious long-term disabilities [1]. One of the disorders that stroke survivors suffer from is hemiplegia, which is the main reason for reduced gait performance [2]. A main feature of the hemiplegic gait is the asymmetric gait pattern, which leads to compensatory patterns such as reduced walking speed and increased risk of falling; and has negative effects on patients’ quality of life [3]. Also, walking independently is an important factor associated with long-term disabilities after stroke, which is considered a therapeutic goal for these patients [4]. Therefore, recovery of gait function is very crucial in stroke rehabilitation [2].

Gait rehabilitation depends on repeated and long-term exercises. Therefore, Robotic-Assisted Gait Training (RAGT) as a new approach is a good suggestion because conventional gait rehabilitation is expensive in various aspects and imposes a lot of burden on the physiotherapist. In addition to reducing the load, the RAGT provides the possibility of frequent, long-term, safe, accurate, personalized, motivational gait exercises with the active participation of patients in various environments and optimal assistance in performing movements [2, 3]. However, systematic reviews acknowledge that wearable lower-limb exoskeletons for gait rehabilitation are in the early stages of development and more Randomized Controlled Trial (RCT) studies are needed to prove their clinical benefits [5–8].

Compared to fixed-type robots, the use of overground robots like Hip Exoskeleton Assistive (HEXA) is more economical and provides more natural sensory inputs in walking by allowing the patient to explore the environment. However, due to the newness of this approach, few studies have been done in this field [9]. However, in some of these studies, the interaction of patients with the robot, regarding the ability to adjust the force, was limited due to either cognitive disorders such as cerebral palsy [10] or complete loss of movement in spinal cord injury patients [8].

Although past studies indicate that patients benefit from robots, there are conflicting results regarding the superiority of this approach over conventional physiotherapy [11]. Some articles have stated that the use of a robot may not have any advantage over conventional gait rehabilitation without a robot, or this type of intervention may not be an effective treatment for improving neuromuscular coordination or balance [2, 6]. Studies also admit that there is a growing need for research on the specific effects of rehabilitation programs based on different types of rehabilitative robots on stroke survivors. This optimizes the use of robots and helps to define standard treatment guidelines [12]. Therefore, according to the above information and the need to know the possible effects of the HEXA robot on the quality of walking, disability, and quality of life of stroke patients, it seems necessary to conduct this study.

The researchers of this study assumed that the use of this robot in gait rehabilitation, compared to conventional physiotherapy, leads to greater improvement in walking quality, disability, and quality of life in stroke patients with hemiplegia.

## Methods

### Participants

All participants provided written informed consent before participation. This study adheres to CONSORT guidelines and it was approved by the ethics committee of Mashhad University of Medical Sciences (IR.MUMS.FHMPM.REC.1400.079 dated 28th Jan 2022). The trial was registered with the clinical trials site of www.IRCT.ir (IRCT20210730052024N1) on January 28th 2022.

All participants were ischemic or hemorrhagic stroke patients in the subacute phase (less than 3 months) [4], diagnosed with hemiplegia and in stage 5 of Brannstrom stages of stroke recovery which is the stage that complex movements return [13]. Considering the assistive approach of the HEXA which is based on the facilitation of patient’s active motions, it was mandatory to participate patients with a minimum strength grade of 3 + in quadriceps and gluteus muscles. In other words, patients should be able to perform active extension in knee and hip joints and weight bearing on the affected limb. Also, all participants aged between 18 and 85 years [14] and they were able to walk without the help of others with or without assistive devices (mild to moderate impairment) [3]. The Miny Mental State Examination (MMSE) score of participants was more than 24 [15] and adequate fit of patients within the exoskeleton (height between 160 and 190 cm and weight between 50 and 100 kg) was considered in this study [2]. The patients with significant musculoskeletal or neurological disorders [4], flaccid paralysis [16], stroke caused by tumor or infection [2], bilateral involvement or quadriplegia [2], inability to walk before stroke [2], leg spasticity more than 3 on Modified Ashworth Scale [2], uncontrolled cardiovascular or respiratory disorders [17], pusher syndrome (i.e., a clinical disorder that causes active pushing away from the non-hemiparetic side in patients with right or left brain damage) [18] and emergence of each exclusion criteria during the implementation of the study were excluded.

### Apparatus

#### Exoskeleton used

**Fig. 1:**
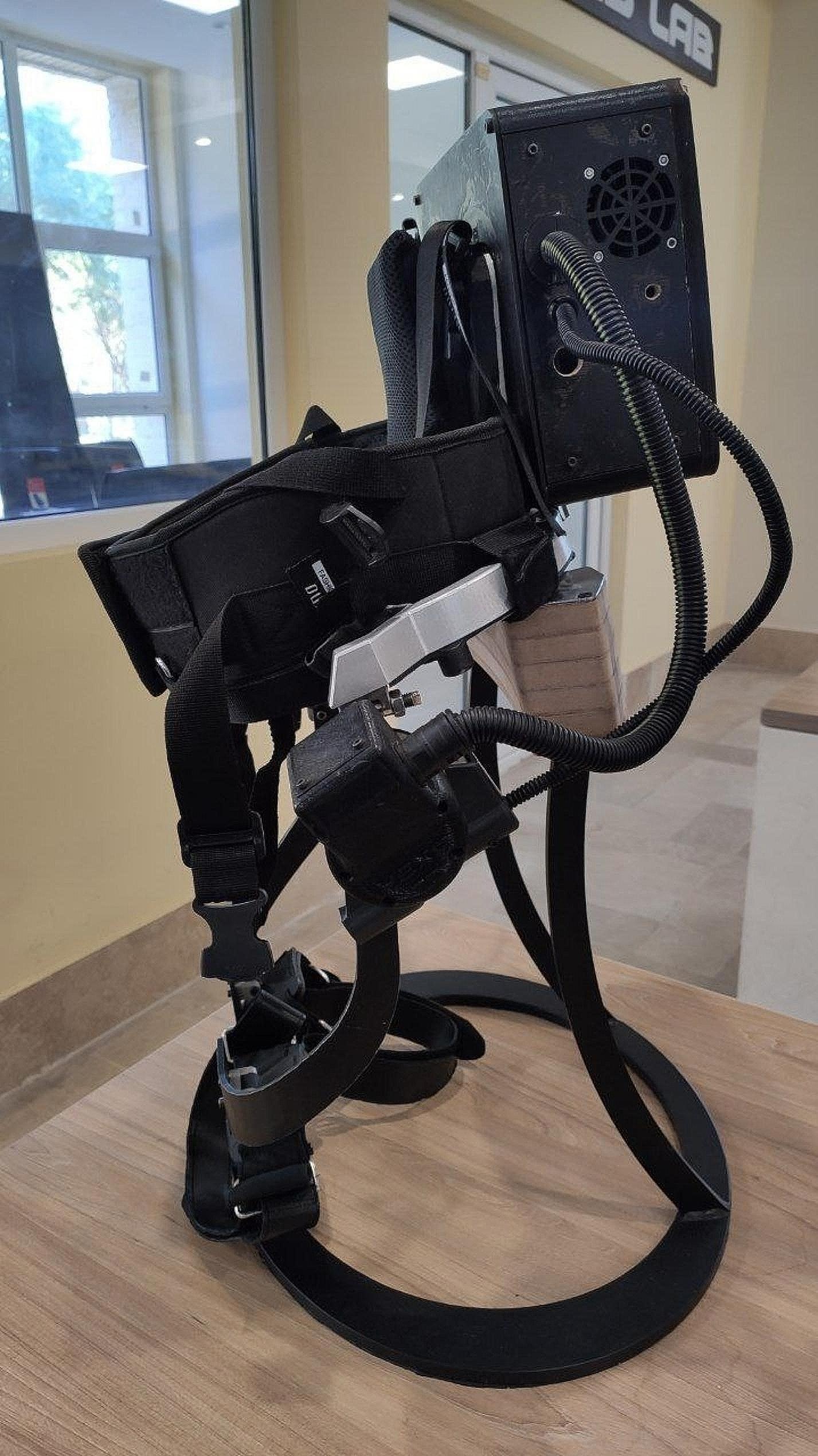
Hip exoskeleton assistive (HEXA) robot

The FUM-HEXA-IV robot (briefly HEXA) is a lower-limb wearable robot that features a single active hip joint. It was designed and built at the Center of Advanced Rehabilitation and Robotics Researches at Ferdowsi University of XXX (FUM-CARE). The robot structure and its actuation mechanism are optimized to provide a high torque capacity while minimizing weight, ensuring that patients experience optimal comfort and the robot is as efficient as possible [10]. The robot weighs approximately 5.5 kg and can generate a maximum torque of 16 N.M. at each hip joint. This is made possible by the use of a 160-watt Maxon EC90 motor and an AG Harmonic Drive gearbox (Fig. 1). The unique feature of this robot is the ability of interaction with the patient which is provided by force sensors, detecting the amount of force generated by the patient and adapting robot’s movements based on the demands of the treatment protocol.

### Clinical outcome measures

6 min Walking Test (6MWT): In 6MWT, the distance covered in 6 min in the unit of meter is used as an indicator of evaluating aerobic capacity. The validity and reliability of this test have been confirmed [19].

Timed Up and Go test (TUG): In TUG, the patient who is sitting stands with the therapist’s signal, moves 3 m forward, then turns around and goes back to the chair and sits and the time of this process in the unit of the second is recorded for assessing the balance in different positions. The validity and reliability of this test have been confirmed [19].

Functional Gait Assessment (FGA): This test is a 10-section tool for measuring walking ability, each section is given from 0 to 3 points (minimum 0 and maximum 30 scores). The Persian version of this tool has been validated [20].

Berg Balance Scale (BBS): This test is a performance-based tool to check functional balance and includes 14 balance sections, each section is given from 0 to 4 points (minimum 0 and maximum 56 scores). The Persian version of this tool has been validated [21].

Stroke Specific-Quality of Life (SS-QOL): This tool is a patient-centered questionnaire that is especially used to evaluate the quality of life of stroke patients. This tool contains 49 sections in different areas related to the quality of life, each section is given from 1 to 5 points (minimum 49 and maximum 245 scores). The Persian version of this questionnaire has been validated [22].

### Procedure

All participants were allocated to two intervention (HEXA) and control groups by randomly blocked allocation method with 6 blocks of 4 subjects. Thus, all 6 permutations of 4 were listed in two groups. The permutations included 1-AABB, 2-ABAB, 3-BAAB, 4-BABA, 5-BBAA, 6-ABBA, and A is the symbol of the intervention group and B is the symbol of the control group. Then, from the table of random numbers with a random starting point, 6 numbers between 1 and 6 were randomly selected by a research assistant, blinded to baseline assessment findings, and according to their order, the patients were assigned to two groups. This study was conducted as a single-blinded pilot RCT, and blinding was done in such a way that one physiotherapist did the evaluation and another physiotherapist did the therapeutic intervention. The duration of treatment in both groups of physiotherapy was 12 sessions, in 3 sessions weekly for 4 consecutive weeks. Before the 1st session and after the 12th session, a physiotherapist other than the therapist (to reduce the bias) evaluated the patients in terms of clinical outcome measures. To reduce the learning and fatigue effects, all the evaluations were done randomly, and in the 1st session, the patient was familiarized with the environment and the tests. Finally, in the statistical software, two study groups were coded as codes 1 and 2, and analysis was done.

**Fig. 2:**
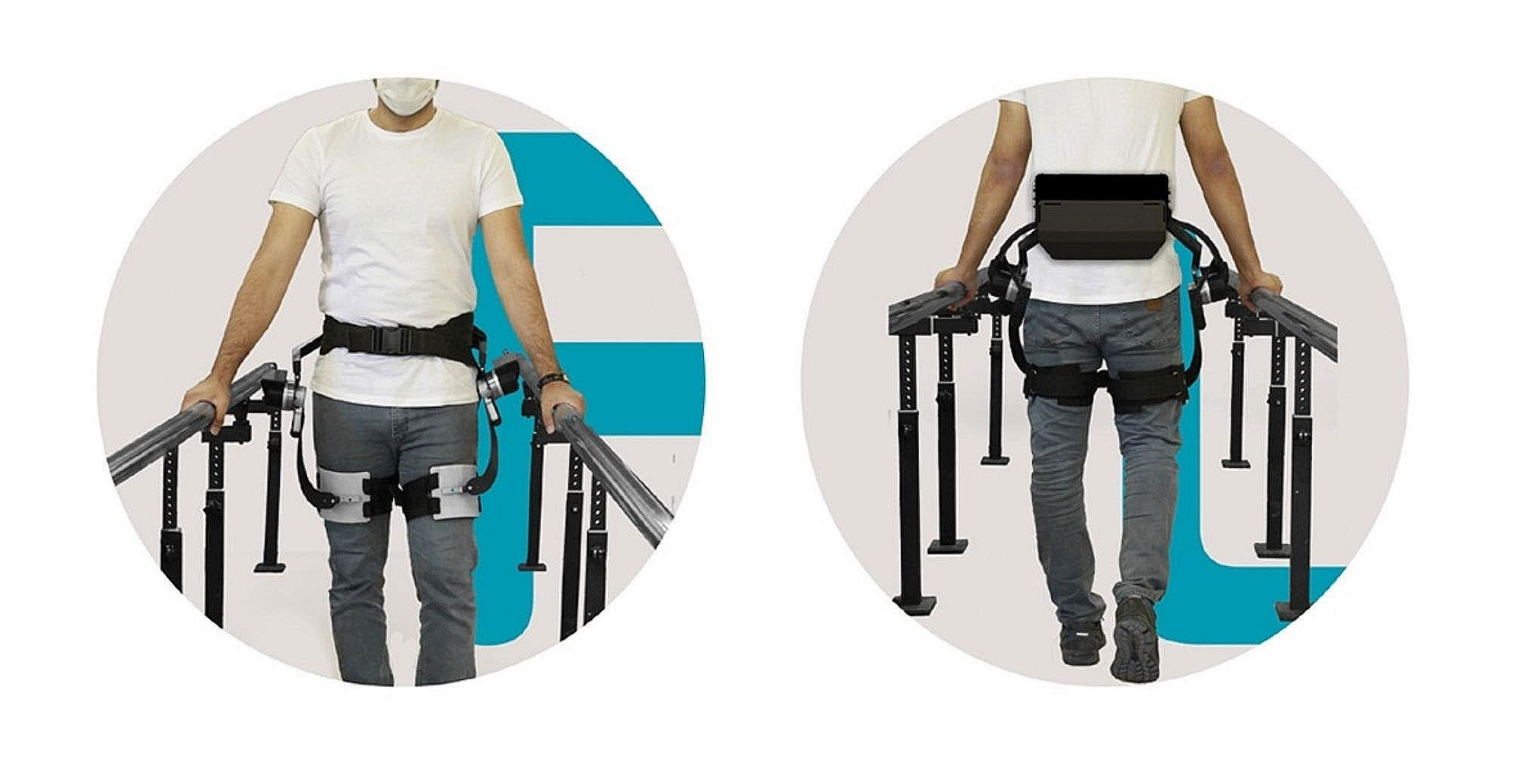
Patients wearing the robot

In both groups, the treatment time for each session was 60 min, including the first 30 min of electrotherapy and conventional exercises for stroke patients, and then 30 min of gait training on the ground. The time required to don and off the robot in the intervention group was not included at this time. Then, in the control group, the patient walked for 30 min with the help of the therapist, and in the HEXA group, the patient placed the robot on the body (Fig. 2) with the help of a therapist and walked for 30 min under the supervision of the therapist [11].

Both groups underwent electrotherapy with Functional Electrical Stimulation current (FES) to contract the ankle dorsiflexor for 15 min. Stimulation parameters included 40 Hz frequency and 0.3 millisecond pulse duration. The intensity was increased until the patient’s tolerance and the on-and-off ratio was 1:1 and according to the comfort of the patient, the same amount of rest was provided for every 5 to 10 s of contraction [23].

As conventional exercises, both groups underwent posture and balance control exercises, weight shifting and active and passive stretching of spastic joints and muscles according to the patient’s tolerance. They also underwent progressive strengthening exercises for weak muscles of the involved limbs and trunk, each exercise 10 to 15 repetitions and in 2–3 sets. Manual resistance was applied by the therapist according to the participants’ capacity [11].

The distance and time of gait training on the ground in the control group were similar to the intervention group, which was done with the help of a therapist and without the assistance of a robot. The physiotherapist reminded the patient through verbal interaction to try to walk as symmetrical as possible [11]. The HEXA consists of two 70-watt BLDC motors with a precision gearbox, each driving a link which is placed on the human thigh and fastened with Velcro straps for either hip. The robot actuators are designed to provide hip flexion torque during the gait cycle. The HEXA has a threshold for the minimum torque of hip flexion and if the patients force reaches that minimum, HEXA acts as an assistive device and just helps the patients to complete the full range of motion. But, if the initial torque of the patient does not reach the distinguished threshold, HEXA would start the motion and based on the participation of the patient, the device would adjust its assistance during the training. The initial settings of the HEXA in the intervention group was as follows: In the first 2 sessions, with 100% power, and from the next sessions, if the conditions and the patient’s power were suitable, we had a 10% decrease in the robot’s help per session with the aim that the patient gains more independence in walking. Whenever the patient needed to rest, the intervention was temporarily stopped and the therapist regularly asked the patient about any pain or discomfort during the exercise. None of the patients in both groups were given any instructions to use their hands while walking, and the patients were free to do so. In both groups, the therapist encouraged the patients to walk as fast as possible and to the extent that their balance was not disturbed. Therefore, by gradually increasing the walking speed according to the patient’s comfort, the intensity of the exercise was proportional to the patient’s physical performance level [3].

### Sample size calculation

By enrolling 12 subjects in each one of the groups, it would be possible to detect a suggested clinically important difference on the 6MWT at an 80% power level with a two-sided alpha of 0.05. Assuming that 20% of patients would be lost to follow-up, the study thus planned to enroll 24 patients that would be assigned to the groups. As the study used repeated measures to analyze the resultant data, providing higher statistical power than t-tests, this was a relatively conservative approach to perform power statistical analysis [24].

### Statistical analysis

Statistical tests were performed using SPSS version 23 with a significant level of *p* < 0.05. Shapiro-Wilk test was used to check the distribution. To check the presence of significant differences between the two groups, an independent t-test was used for the Body Mass Index (BMI) variable, and the Mann-Whitney test was used for age and mental state variables. To check the presence of significant differences between the two groups, Pearson’s chi-squared test was used for the qualitative variables of gender, lesion type, involved side, and hypertension, and the Likelihood ratio test was used for the variable of diabetes.

To analyze the clinical outcomes, a 2 × 2 Analysis of Variance (ANOVA) with the within-subject factor of time and the between-subject factor of the group was used. Due to the non-normal distribution, the outcome data of 6MWT were first transformed into a logarithm at base 10 and then analyzed with ANOVA. The results of TUG and BBS, which had normal distribution in one group and non-normal distribution in the other group, were analyzed with both ANOVA without logarithmic transformation and ANOVA after logarithmic transformation. Since the results were the same, for the sake of brevity, only the results of ANOVA without logarithmic transformation were provided [25].

## Results

**Fig. 3:**
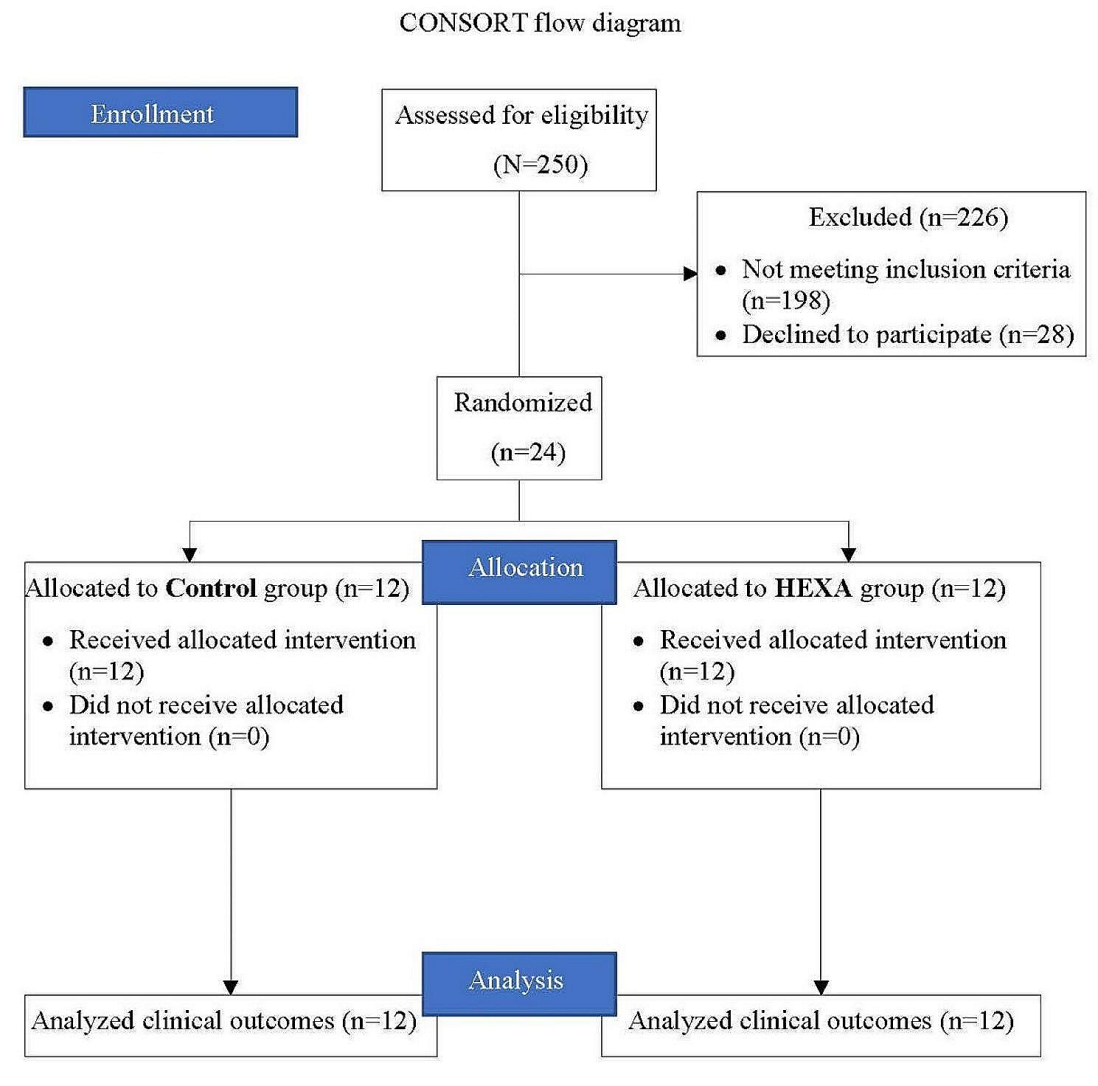
Flowchart of the study

Totally 24 patients out of 250 patients were enrolled in the study (the CONSORT diagram is shown in Fig. 3). In terms of demographic characteristics, there was no statistically significant difference between the groups (Table 1).

**Table 1:** Demographic and clinical characteristics of the participants (*N* = 24)

Variable	Intervention (*n* = 12)	Control (*n* = 12)	*P* value
**Age (year)**	46.58 (15.58)	53.33 (9.19)	0.112
**BMI (kg/cm** ^**2**^ **)**	25.60 (2.42)	24.26 (2.96)	0.238
**MMSE**	29.33 (0.88)	29.58 (0.66)	0.525
**Gender (male : female)**	7 : 5	5 : 7	0.414
**Lesion type (ischemic : hemorrhagic)**	7 : 5	3 : 9	0.098
**Involved side (left : right)**	8 : 4	5 : 7	0.219
**Hypertension**	6	5	0.682
**Diabetes**	4	3	0.653

Age, BMI and MMSE are expressed as mean (SD). Significancy is defined *p* < 0.05

BMI = Body Mass Index. MMSE = Mini-Mental State Examination

The interaction of group by time was only significant for the BBS variable [F [1] = 28.05, *P* = 0.000] (Table 2). Therefore, the analysis of the group within time was performed and the Wilcoxon test showed that both groups significantly improved at post-test compared to pre-test (*P* = 0.005 for intervention group and *P* = 0.002 for control group).

**Table 2:** Changes in clinical outcomes with ANOVA

Outcome	Intervention group		Control group		Main effect				Interaction effect	
					Time		Group		Time × group	
	Pre	Post	Pre	Post	*P*-Value	F-Ratio	*P*-Value	F-Ratio	*P*-Value	F-Ratio
**6MWT(m)**	139.55 (86.45)	230.57 (119.72)	71.39 (25.12)	119.51 (48.99)	**0.000**	83.729	**0.005**	9.588	0.931	0.008
**TUG(s)**	31.32 (19.17)	19.15 (11.73)	45.20 (13.33)	29.74 (7.68)	**0.000**	56.415	**0.029**	5.430	0.379	0.805
**FGA (score)**	15.33 (7.97)	22.67 (5.49)	10.42 (4.85)	19.25 (4.65)	**0.000**	91.189	0.078	3.422	0.385	0.785
**BBS (score)**	43.92 (9.99)	48.67 (6.47)	28.75 (8.57)	46.08 (5.01)	**0.000**	86.416	**0.006**	9.151	**0.000**	28.058
**SS-QOL (score)**	148.50 (32.82)	159.58 (41.44)	131.58 (24.19)	155.17 (27.51)	**0.004**	10.614	0.384	0.790	0.253	1.380

All outcomes are expressed as mean (SD). Bold numbers indicate significancy (*p* < 0.05)

6MWT(m) = 6 min Walking Test in unit of meter. TUG(s) = Timed Up and Go in unit of second. FGA = Functional Gait Assessment. BBS = Berg Balance Scale. SS-QOL = Stroke Specific Quality of Life

The main effects of time and group were significant for the 6MWT (*P* = 0.000 and *P* = 0.005, respectively) and TUG (*P* = 0.000 and *P* = 0.029, respectively). In other words, there were significant differences within the groups (post compared to pre) and between the groups (intervention compared to control) in these two outcomes (Table 2).

The main effect of time was significant for the FGA and SS-QOL (*P* = 0.000 and *P* = 0.004, respectively). Both groups had significant differences at post compared to pre, but there were no significant differences between the groups (*P* = 0.078 and *P* = 0.384, respectively) (Table 2).

## Discussion

This pilot RCT was conducted to investigate the effect of using the HEXA robot on the quality of walking, disability, and quality of life of stroke patients with hemiplegia compared to conventional physiotherapy. The results showed that both groups significantly improved in all outcomes post-intervention compared to pre-intervention time. Also, compared to the control group, the participants in the HEXA group on average covered more distance in the 6MWT test and completed the TUG test faster.

The first domain of the International Classification of Functioning, Disability and Health (ICF) is the functional and structural impairments of the body. Based on this study, it is evident that short-term rehabilitation interventions can effectively improve functional and structural impairments. For instance, the main effect of the group was significant in 6MWT and TUG which are related to walking quality. On the other hand, the main effect of the group was not significant in FGA and SSQOL related to disability and quality of life. However, to effectively improve activity limitations and participation restrictions at the next domains of ICF, we need comprehensive, long-term rehabilitation programs and cooperation between different disciplines such as rehabilitation, medicine, and engineering. The ICF is a classification system and is a foundation for understanding the patient’s personal and environmental resources and limitations. The classification system identifies three domains of a health condition: [1] body function (physiological and psychological) and structure (related to organs, limbs, etc.) [2], activity (related to the execution of a task, and [3] participation (related to involvement in a real-life situation) [6].

The results obtained in the present study are consistent with most of the results obtained by Jayaraman et al. (2019), in which significant improvements in 6MWT, FGA, BBS, and SS-QOL outcomes were observed within the groups. In terms of between-group differences, in 6MWT and BBS outcomes, there was a significantly greater improvement in the robotic group [14]. On the other hand, Lee et al. (2019) did not observe any significant differences in the BBS outcome within and between groups [3]. Also, Yoo et al. (2023) found a significant improvement in the BBS outcome in both groups, but there was no significant difference between the groups. Also, the TUG outcome was significantly improved only in the robotic group [9]. In the study of Miyagawa et al. (2023), despite improvement in both groups, no significant differences were observed between the two groups in 6MWT, TUG, and BBS outcomes [26]. Comparing the results of the present research with the aforementioned studies and the lack of consistency in some results shows that depending on the difference in the parameters affecting the research, such as the type of method, the type of robot, the phase of the disease, the number and duration of treatment sessions, etc., we will see different results. Therefore, we need more comprehensive research to clarify all aspects of the advantages and disadvantages of robotic rehabilitation approaches.

This study has limitations, the main of which include the small sample size, lack of follow-up period, and lack of information about the placebo effect of the HEXA robot.

To continue working in this area, it is suggested that studies with larger sample size and mid and long-term follow-up periods be conducted so that the results can be more reliably generalized to the target population. It is also suggested that studies be designed to measure the placebo effect of the robot to prove whether this type of intervention is a real treatment. In addition, considering that there is still no consensus on the optimal use of robots and their intervention parameters such as the intensity and time of assistance and the assistance strategy used; analyzing these issues on the HEXA robot will be very important for further research in the future [7].

In conclusion, it seems that the HEXA robot can effectively improve walking capacity by over 60% and speed by 30%. Considering its therapeutic benefits and taking into account the difficult and time-consuming nature of conventional gait rehabilitation, it seems that this robot can play a role as an effective rehabilitation tool by providing frequent and targeted walking exercises.

### Electronic supplementary material

Below is the link to the electronic supplementary material.

Supplementary Material 1

## Data Availability

The datasets used and/or analyzed during the current study are available from the corresponding author on reasonable request.
